# Weinreb Amidation as the Cornerstone of an Improved Synthetic Route to A-Ring-Modified Derivatives of Luotonin A

**DOI:** 10.3390/molecules171011363

**Published:** 2012-09-25

**Authors:** Norbert Haider, Simon Nuß

**Affiliations:** Department of Drug and Natural Product Synthesis, Faculty of Life Sciences, University of Vienna, Althanstraße 14, A-1090 Vienna, Austria

**Keywords:** Weinreb amidation, luotonin A, [4+2] cycloaddition, thiophene

## Abstract

Weinreb amidation of ethyl 4-*oxo*-3,4-dihydroquinazoline-2-carboxylate with aromatic amines provides a significantly improved route to anilide-type key intermediates for the synthesis of the anticancer alkaloid, luotonin A, and new A-ring-modified derivatives thereof. This method has advantages concerning overall yield, brevity, and versatility with regard to the aromatic amine component, even if the latter has less favourable nucleophilicity, solubility and/or stability properties. This is demonstrated by the concise synthesis of a small library of luotonin A analogues, including a novel thiophene isostere of the alkaloid.

## 1. Introduction

**Figure 1:**
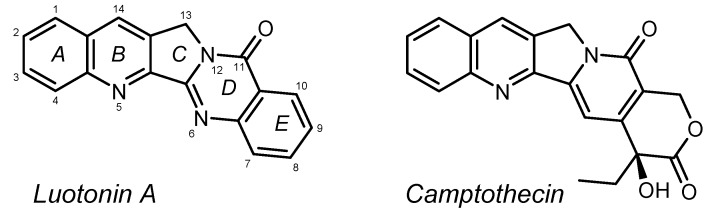
Structures of luotonin A (with IUPAC-conforming numbering) and camptothecin.

Since the isolation of the alkaloid luotonin A (Figure 1) from the plant *Peganum nigellastrum* Bunge (Zygophyllaceae) in 1997 [1] and the discovery of its cytotoxic activity, which is based on stabilisation of the DNA-topoisomerase-I complex and is thus similar to that of the closely related natural product, camptothecin (CPT; Figure 1), there have been various reports describing the total synthesis and biological evaluation of luotonin A and compounds derived thereof (for reviews on luotonin A and related natural products, see refs. [2,3,4,5]). These synthetic approaches include the assembly of a pyrroloquinoline fragment (rings ABC) with an anthranilic acid synthon [6] as well as free-radical cyclisation reactions [7,8] or Pd-catalysed CC coupling reactions [9] as the key step, the latter usually with final formation of ring C (for a comprehensive overview of all the synthetic strategies employed so far, cf. ref. [5]).

**Scheme 1:**
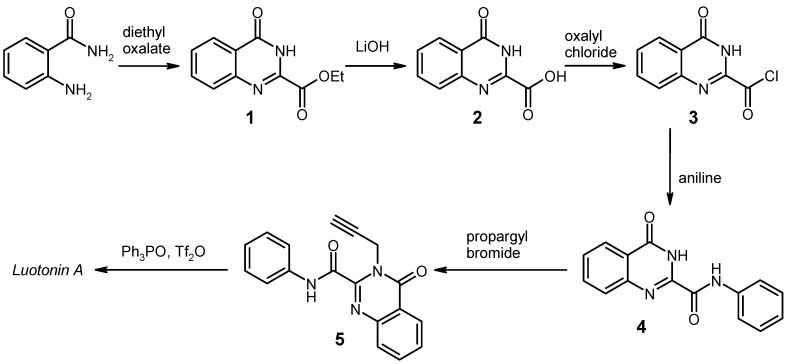
Yao’s total synthesis of luotonin A [12].

As it has become quite evident that modification of ring A is highly interesting with respect to achieving enhanced cytotoxicity [5], synthetic approaches which utilise easily accessible and highly variable A-ring building blocks are of particular value. In this context, three different methods employing aniline or substituted anilines as such synthons have been developed. In Batey’s approach [10], the key step is an intramolecular aza-Diels-Alder reaction of an imine-containing azadiene with a propargyl dienophile, mediated by dysprosium triflate (an application of the Povarov reaction). Closely related is a one-pot reaction recently published by Chu [11] which gives luotonin A or some derivatives thereof, albeit in modest yields, from isatoic anhydride, propargylamine, glyoxal and anilines in the presence of ytterbium triflate. In our hands, the cycloaddition approach reported by Yao [12] (Scheme 1) gave the highest overall yields and it worked most reliably in the final cycloaddition key step.

This route is based on the generation of the requisite azadiene from an anilide structure **5** by treatment with Hendrickson’s reagent [bis(triphenyl)oxodiphosphonium trifluoromethanesulfonate, prepared *in situ* from triphenylphosphine oxide and trifluoromethanesulfonic anhydride]. In Yao’s protocol [12], the intermediate anilide **4** is prepared by saponification of ethyl 4-*oxo*-3,4-dihydro­quinazoline-2-carboxylate (**1**) [13,14,15] with lithium hydroxide in aqueous tetrahydrofuran, followed by treatment of the carboxylic acid **2** with oxalyl chloride and subsequent reaction of the acid chloride **3** thus obtained with aniline/sodium bicarbonate in dichloromethane.

## 2. Results and Discussion

When we sought to employ this protocol for the preparation of a small library of new luotonin A derivatives bearing various substituents at ring A, we soon found out that the sequence of ester hydrolysis/acid chloride formation/aminolysis constitutes a serious bottleneck for two reasons: (a) the free carboxylic acid **2** is almost insoluble in most solvents, but it is highly sensitive towards decarboxylation, and special care has to be taken during the preparation and work-up of this intermediate; (b) the reaction of the very sparingly soluble acid chloride **3** with aromatic amines can also be problematic, especially if they have less favourable properties than aniline in terms of nucleophilicity, solubility, and/or stability. In order to overcome these limitations, we have now developed a highly efficient alternative route for the preparation of these key intermediates which enabled us to synthesize a series of A-ring modified luotonin A derivatives, including a novel thiophene analogue of the lead compound.

**Scheme 2:**
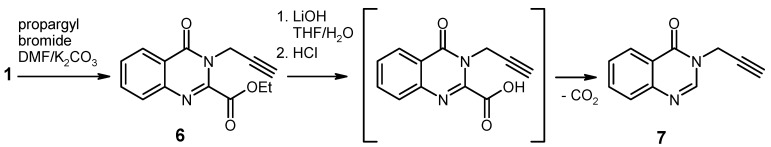
Hydrolysis of the ester **6**, followed by spontaneous decarboxylation.

In a first attempt to circumvent the solubility problems mentioned above, we reversed the order of the two steps, *N*-alkylation (with propargyl bromide) and ester hydrolysis (Scheme 2). However, this approach met with failure: hydrolysis of the *N*-propargyl ester **6** with lithium hydroxide in aqueous tetrahydrofuran at room temperature or below always resulted in immediate decarboxylation of the initially formed carboxylic acid upon neutralisation (pH 7), leading to the 2-unsubstituted quinazolinone **7**, which is known from literature [13,14,15].

**Scheme 3:**
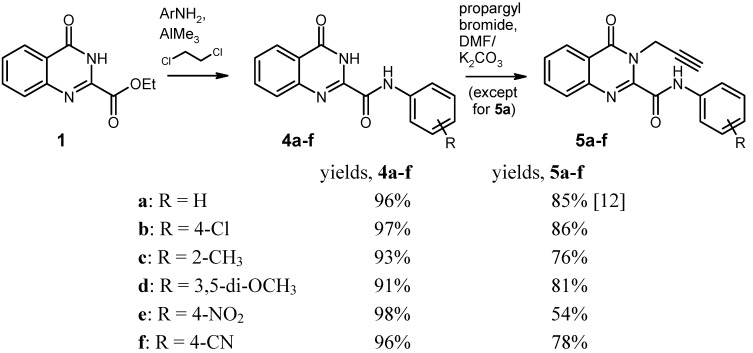
Two-step synthesis of the key intermediates **5** from the ester **1**.

Consequently, we envisaged direct transformation of the ester function into the corresponding anilide as a general and versatile solution of this problem. Weinreb’s method [16,17] for amide synthesis from esters and (aliphatic or aromatic) amines by activation of the amine component with trimethylaluminium appeared to be an attractive option. Indeed, when the *N*-propargyl ester **6** was reacted with AlMe_3_-activated aniline in 1,2-dichloroethane solution (this solvent had been reported to be superior [18]), slow formation of the anilide was detected by TLC. However, the reaction could not be brought to completion even after prolonged refluxing and by using a larger excess of reagent. We assume steric hindrance of the ester group by the adjacent propargyl moiety to be responsible for this insufficient reactivity. Therefore, the *N*-3 unsubstituted ester **1** was chosen as a more promising substrate for Weinreb amidation. It turned out that this approach indeed gives excellent results: typically, **1** is completely consumed within 1-2 hours of refluxing, and the corresponding anilides **4** are obtained in >90% yield (Scheme 3). This applies even to sparingly soluble and/or electron-poor anilines like 4-nitroaniline or 4-aminobenzonitrile. After quenching of the amidation reactions with aqueous acid, the products **4** are typically isolated simply by filtration.

For the subsequent N-alkylation step with propargyl bromide, we again had to modify Yao’s protocol [12] in order to circumvent solubility problems with some of the substituted anilides **4**. Instead of the liquid/liquid two-phase method (toluene/water, tetrabutylammonium bromide as phase-transfer catalyst), we used dimethyl formamide/potassium carbonate as a reaction medium, which reliably gave the products (**5**) in yields between 54% and 86% (Scheme 3).

**Scheme 4:**
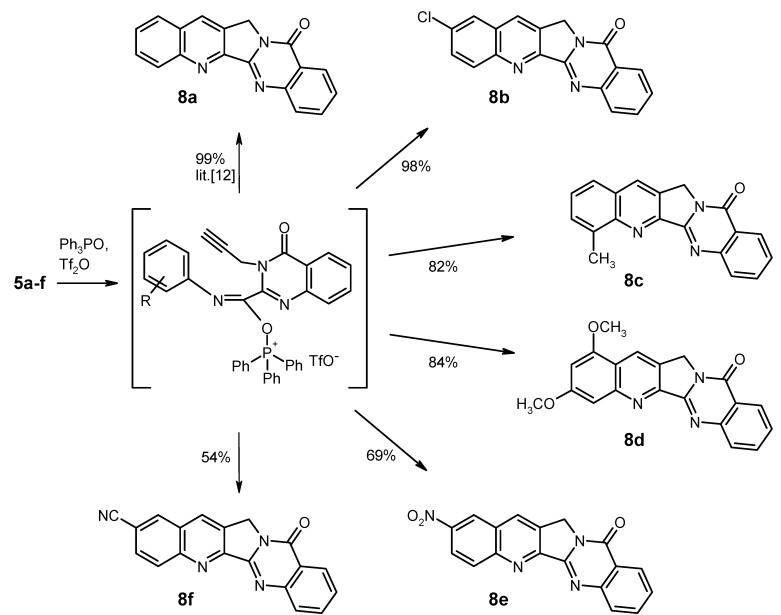
Synthesis of luotonin A derivatives by intramolecular cycloaddition of compounds **5**.

With this optimised pathway to the 3-substituted quinazolinone-2-carboxylic acid anilides **5** now available, we prepared several of these cycloaddition educts and subjected them to the cycloaddition conditions [12] (triphenylphosphine oxide, triflic anhydride, dry dichloromethane as solvent; see Scheme 4). In most cases, the intramolecular aza-Diels-Alder reaction takes place very smoothly at room temperature within one hour. Thus, the known compounds luotonin A (**8a**) [1], 2-chloroluotonin A (**8b**) [19], and 4-methylluotonin A (**8c**) [11] as well as the new analogue 1,3-dimethoxyluotonin A (**8d**) were prepared in high yields (82–99%). When the aniline unit bears a strongly electron-withdrawing and solubility-decreasing group, as it is the case with the 4-nitroanilide **5e** and the 4‑cyanoanilide **5f**, the cycloaddition proceeds somewhat less efficiently, affording the hitherto unknown 3-nitroluotonin A (**8e**) and 3-cyanoluotonin A (**8f**) in 69% and 54% yield, respectively, along with some unreacted educt [20].

**Scheme 5:**
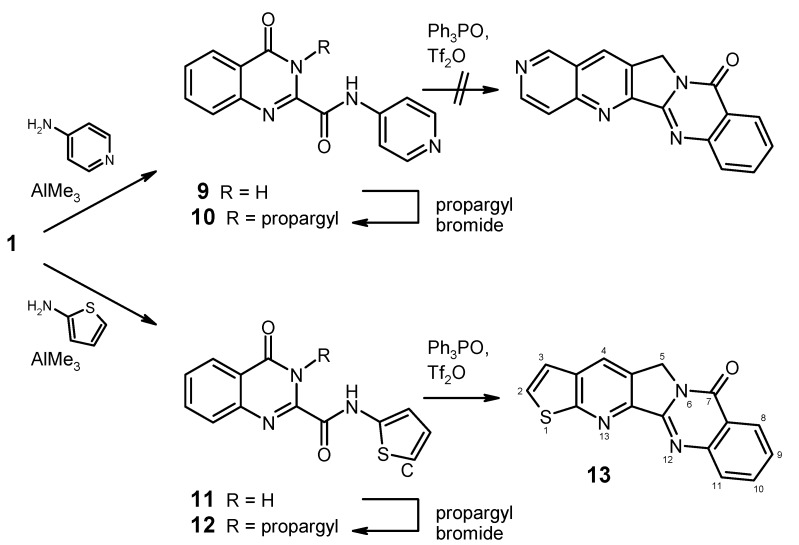
Preparation and (attempted) cyclisation of heterocyclic analogues.

In order to further explore the scope and limitations of this concise route to luotonin A derivatives, we briefly investigated its suitability for generating heterocyclic A-ring analogues, using 4-amino­pyridine and 2-aminothiophene as building blocks. While the former is commercially available and undergoes Weinreb amidation with **1** very smoothly to afford the *N*-(4-pyridyl)amide **9** (Scheme 5), the latter amine is rather unstable and rapidly decomposes after isolation as the free base. We used a modification of Binder’s method [21] for the preparation of 2-aminothiophene by deprotection of *N*‑BOC-2-aminothiophene, employing a mixture of dichloromethane and trifluoroacetic acid at 0 °C. Instead of attempting to isolate the free amine, we simply washed the organic solution with aqueous sodium carbonate and subsequently performed a solvent exchange from dichloromethane to 1,2‑dichloroethane, keeping the temperature below 20 °C all the time. After drying, this solution of 2‑aminothiophene is well-suited for use in the Weinreb amidation, thus affording the *N*‑(2‑thienyl)amide **11** in 95% yield (Scheme 5).

Both of the *N*-(hetaryl)amides **9**,**11** were selectively alkylated at the quinazolinone nitrogen with propargyl bromide in analogy to the preparation of compounds **5**. However, attempted intramolecular cycloaddition by treatment with Hendrickson’s reagent showed that the pyridine derivative **10** does not react under these conditions: only unchanged starting material and triphenylphosphine oxide were recovered (a tiny TLC spot with an intense blue fluorescence indicated that traces of the 2-azaluotoninA might have been formed, though). On the other hand, we succeeded in the transformation of the thiophene intermediate **12** into the desired pentacyclic compound **13** in reasonable yield (55%), the latter product representing a novel thiophene isoster of luotonin A [22].

Preliminary *in-vitro* screening (using the Resazurin assay [23]) for antiproliferative activity towards six different human tumor cell lines showed that **13** is practically inactive, whereas **8d** exhibits a slightly better activity profile than that of the lead compound, luotonin A [24]. More detailed biological investigations are intended.

## 3. Experimental

### General

Melting points (uncorrected) were determined on a Kofler hot-stage microscope (Reichert). ^1^H‑NMR and ^13^C-NMR spectra were recorded on a Varian Unity*Plus* 300 spectrometer at 300 MHz and 75 MHz, respectively. IR spectra were taken on a Perkin-Elmer 1605 FT-IR instrument. Mass spectra were obtained on a Shimadzu QP5050A DI 50 instrument, high-resolution mass spectra were recorded on a Finnigan MAT 8230 spectrometer at the Institute of Organic Chemistry, University of Vienna. Column chromatography was carried out on Merck Kieselgel 60, 0.063–0.200 mm, thin layer chromatography was done on Merck aluminium sheets pre-coated with Kieselgel 60 F_254_. Microanalyses were performed at the Microanalytical Laboratory, Faculty of Chemistry, University of Vienna. Compounds **5a** and **8a** were prepared according to lit.[12].

*Ethyl 4-oxo-3-(prop-2-yn-1-yl)-3,4-dihydroquinazoline-2-carboxylate* (**6**). To a solution of ethyl 4-*oxo*-3,4-dihydroquinazoline-2-carboxylate [13,14,15] (**1**, 1.004 g, 4.6 mmol) in dimethylformamide (20 mL) was added K_2_CO_3_ (759 mg, 5.5 mmol) and propargyl bromide (818 mg of an 80% solution in toluene, 5.5 mmol), and the mixture was stirred at room temperature for 4 h. It was then poured into ice-water (50 mL) and extracted with ethyl acetate (3 × 50 mL). The combined extracts were washed with brine, dried over Na_2_SO_4_ and evaporated under reduced pressure to afford an oily residue. Trituration with cold ethanol gave the product (1.110 g, 94%) as colourless crystals, mp 93–94 °C (EtOH). MS (EI, 70 eV) *m/z*: 256 (M^+^, 88%), 227 (100), 184 (74), 183 (44), 155 (75), 130 (46), 129 (69), 102 (41); ^1^H‑NMR (CDCl_3_) δ: 8.34 (d, *J* = 7.9 Hz, 1H, 5-H), 7.86–7.76 (m, 2H, 7-H, 8-H), 7.63–7.54 (m, 1H, 6-H), 5.17 (d, *J* = 2.4 Hz, 2H, NCH_2_), 4.56 (q, *J* = 7.2 Hz, 2H, OCH_2_CH_3_), 2.32 (t, *J* = 2.4 Hz, 1H, C≡CH), 1.49 (t, *J* = 7.2 Hz, 3H, OCH_2_CH_3_); ^13^C-NMR (CDCl_3_) δ: 161.3, 160.5, 146.1, 145.6, 135.0, 128.9, 128.5, 127.4, 121.9, 73.3, 63.7, 33.3, 14.1. Anal. Calcd. for C_14_H_12_N_2_O_3_: C, 65.62; H, 4.72; N, 10.93. Found: C, 65.63; H, 4.74; N, 10.82.

*Hydrolysis/Decarboxylation of the Ester* **6**. To an ice-cooled solution of the ester **6** (512 mg, 2 mmol) in THF (10 mL) was added water (3 mL) and LiOH monohydrate (126 mg, 3 mmol) and the mixture was stirred at 0 °C for 10 min. The solution was concentrated under reduced pressure to approx. 3 mL and it was then diluted with ice-cold water (7 mL), neutralised with 1 N HCl, and extracted with ethyl acetate (3 × 20 mL). The combined extracts were washed with brine, dried over Na_2_SO_4_ and evaporated under reduced pressure to give *3-(prop-2-yn-1-yl)quinazolin-4(3H)-one* [13] (**7**, 287 mg, 93%) as an oily residue which slowly solidified; recrystallisation from methanol afforded colourless crystals of mp 115–116 °C (lit. [13] mp 116–118 °C, lit. [14] mp 115–116 °C). ^1^H-NMR (CDCl_3_) δ: 8.35–8.26 (m, 2H, 2-H, 5-H), 7.82–7.69 (m, 2H, 7-H, 8-H), 7.51 (dd, *J* = 6.5 Hz, 1.9 Hz, 1H, 6-H), 4.81 (d, *J* = 2.5 Hz, CH_2_), 2.49 (t, *J* = 2.5 Hz, 1H, C≡CH).

*General Procedure for the Synthesis of the Anilides ***4*** by Weinreb Amidation.* To a solution of the corresponding aniline (8 mmol) in dry 1,2-dichloroethane (20 mL) under argon was added dropwise a 2 M solution of AlMe_3_ (4.0 mL, 8 mmol) in heptane. The mixture was stirred for 30 min at room temperature, then the ester **1** (1.091 g, 5 mmol) was added in one portion, and the mixture was refluxed for 2 h. After cooling to 0 °C, it was then quenched by slow addition of 2 N HCl (20 mL), followed by water (80 mL). The resulting liquid/liquid/solid system was filtered and the filter cake was washed with 70% EtOH and dried. An additional amount of product was obtained by exhaustive extraction of the filtrate with CH_2_Cl_2_, washing of the extract with water, drying over Na_2_SO_4_ and evaporation. The combined portions of crude product were recrystallised from an appropriate solvent (see below).

*4-Oxo-N-phenyl-3,4-dihydroquinazoline-2-carboxamide* [12,25] (**4a**). Yield: 1.240 g (93%), colourless crystals, mp 247–248 °C (EtOH) (lit. [25] mp 178 °C). MS (EI, 70 eV) *m/z*: 265 (M^+^, 47%), 223 (11), 146 (100), 119 (67), 118 (15), 91 (15), 90 (20), 77 (8), 65 (12); ^1^H-NMR (DMSO-*d*_6_) δ: 12.50 (br s, 1H, 3-H), 10.76 (s, 1H, amide-H), 8.20 (dd, *J* = 7.7 Hz, 1.1 Hz, 1H, 5-H), 7.94–7.85 (m, 4H, 7-H, 8-H, phenyl 2′-H, 6′-H), 7.65–7.61 (m, 1H, 6-H, shows NOE on irradiation at 8.20 ppm), 7.39 (t, *J* = 7.8 Hz, 2H, phenyl 3′-H, 5′-H), 7.19–7.14 (m, 1H, phenyl 4′-H); ^13^C-NMR (DMSO-*d*_6_) δ: 161.1, 158.1, 146.8, 146.1, 137.7, 134.8, 128.8, 128.2, 127.7, 126.2, 124.6, 122.7, 120.6.

*N-(4-Chlorophenyl)-4-oxo-3,4-dihydroquinazoline-2-carboxamide* (**4b**). Yield: 1.450 g (97%), colourless crystals, mp 294–295 °C (DMF/CHCl_3_). IR (KBr): 3322, 3232, 3136, 3116, 1714, 1706, 1696, 1684, 1590, 1540, 1492, 1402, 1312, 840, 816, 768, 496 cm^-1^; MS (EI, 70 eV) *m/z*: 301 (M^+^, 13%), 300 (7), 299 (M^+^, 38), 146 (48), 119 (100), 91 (11), 90 (21), 63 (10); ^1^H-NMR (DMSO-*d*_6_) δ: 12.52 (s, 1H, 3-H), 10.93 (s, 1H, amide-H), 8.19 (d, *J* = 7.2 Hz, 1H, 5-H), 7.94–7.87 (m, 4H, 7-H, 8-H, phenyl 2′-H, 6′-H, shows NOE on irradiation at 10.93 ppm), 7.66–7.61 (m, 1H, 6-H, shows NOE on irradiation at 8.19 ppm), 7.48–7.43 (AA′ part of an AA′BB′ system, 2H, phenyl 3′-H, 5′-H); ^13^C-NMR (DMSO-*d*_6_) δ: 161.1, 158.2, 146.7, 146.0, 136.6, 134.7, 128.6, 128.3, 128.2, 127.6, 126.2, 122.7, 122.2. HRMS (EI, 70 eV) *m/z* calcd. for C_15_H_11_ClN_3_O_2_ ([M+H]^+^): 300.0540. Found: 300.0550.

*N-(2-Methylphenyl)-4-oxo-3,4-dihydroquinazoline-2-carboxamide* (**4c**). Yield: 1.298 g (93%), colourless crystals, mp 215–217 °C (DMF/CHCl_3_). IR (KBr): 3350, 3132, 3062, 1678, 1604, 1590, 1542, 1444, 1334, 896, 772, 668 cm^-1^; MS (EI, 70 eV) *m/z*: 279 (M^+^, 35%), 251 (44), 146 (44), 120 (28), 119 (100), 118 (22), 91 (20), 90 (27); ^1^H-NMR (DMSO-*d*_6_) δ: 12.51 (s, 1H, 3-H), 10.36 (s, 1H, amide-H), 8.19 (dd, *J* = 8.1 Hz, 1.2 Hz, 1H, 5-H), 7.94–7.83 (m, 2H, 7-H, 8-H), 7.86–7.60 (m, 2H, 6-H, phenyl 6′‑H, shows NOE on irradiation at 10.36 ppm or at 8.19 ppm), 7.31–7.25 (m, 1H, phenyl 3′-H), 7.25-7.14 (m, 2H, phenyl 4′-H, 5′-H), 2.31 (s, 3H, CH_3_); ^13^C-NMR (DMSO-*d*_6_) δ: 161.2, 157.8, 146.6, 146.0, 135.1, 134.7, 131.5, 130.4, 128.1, 127.6, 126.2, 126.1, 125.9, 124.1, 122.6, 17.5. HRMS (EI, 70 eV) *m/z* calcd. for C_16_H_14_N_3_O_2_ ([M+H]^+^): 280.1086. Found: 280.1089.

*N-(3,5-Dimethoxyphenyl)-4-oxo-3,4-dihydroquinazoline-2-carboxamide* (**4d**). Yield: 1.480 g (91%), colourless crystals, mp 228–229 °C (CHCl_3_). IR (KBr): 3334, 3130, 3076, 3002, 2938, 1672, 1600, 1548, 1466, 1444, 1416, 1334, 1200, 1152, 1064, 1056, 892, 812, 776, 680, 566 cm^-1^; MS (EI, 70 eV) *m/z*: 326 (14%), 325 (M^+^, 100), 310 (16), 294 (43), 283 (12), 179 (17), 146 (36), 119 (71), 90 (22); ^1^H‑NMR (DMSO-*d*_6_) δ: 12.49 (s, 1H, 3-H), 10.67 (s, 1H, amide-H), 8.19 (d, *J* = 9.6 Hz, 1H, 5-H), 7.94–7.87 (m, 2H, 7-H, 8-H), 7.66–7.60 (m, 1H, 6-H), 7.19 (d, *J* = 2.1 Hz, 2H, phenyl 2′-H, 6′-H, shows NOE on irradiation at 10.67 ppm), 6.32 (t, *J* = 2.1 Hz, 1H, phenyl 4′-H) 3.75 (s, 6H, OCH_3_); ^13^C-NMR (DMSO-*d*_6_) δ: 161.1, 160.4, 158.0, 146.6, 145.9, 139.2, 134.7, 128.2, 127.6, 126.2, 122.6, 98.7, 96.6, 55.2. HRMS (EI, 70 eV) *m/z* calcd. for C_17_H_16_N_3_O_4_ ([M+H]^+^): 326.1141. Found: 326.1145.

*N-(4-Nitrophenyl)-4-oxo-3,4-dihydroquinazoline-2-carboxamide* (**4e**). Yield: 1.521 g (98%), colourless crystals, mp > 330 °C (DMF). IR (KBr): 3292, 3254, 3116, 1718, 1698, 1606, 1554, 1514, 1484, 1450, 1412, 1336, 1308, 1232, 1184, 1146, 1108, 994, 898, 858, 770, 750, 718, 686, 664, 594, 546, 526, 490 cm^−1^; MS (EI, 70 eV) *m/z*: 310 (M^+^, 57%), 146 (100), 145 (15), 119 (100), 118 (19), 91 (16), 90 (28), 57 (15); ^1^H-NMR (DMSO-*d*_6_) δ: 12.63 (s, 1H, 3-H), 11.33 (s, 1H, amide-H), 8.32–8.28 (BB′ part of an AA′BB′ system, 2H, phenyl 3′-H, 5′-H), 8.23–8.16 (m, 3H, 5-H, phenyl 2′-H, 6′-H, shows NOE on irradiation at 11.33 ppm), 7.97–7.88 (m, 2H, 7-H, 8-H), 7.69–7.63 (m, 1H, 6-H); ^13^C-NMR (DMSO-*d*_6_) δ: 160.9, 158.8, 146.7, 145.5, 143.8, 143.2, 134.8, 128.5, 127.9, 126.2, 124.7, 122.9, 120.5. HRMS (EI, 70 eV) *m/z* calcd. for C_15_H_10_N_4_O_4_ (M^+^): 310.0702. Found: 310.0710.

*N-(4-Cyanophenyl)-4-oxo-3,4-dihydroquinazoline-2-carboxamide* (**4f**). Yield: 1.392 g (96%), colourless crystals, mp 322–324 °C (DMF). IR (KBr): 3304, 3256, 2224, 1720, 1688, 1604, 1590, 1536, 1484, 1466, 1446, 1412, 1320, 1232, 1176, 1142, 1120, 896, 840, 770, 710, 684, 600, 550, 506, 460 cm^−1^; MS (EI, 70 eV) *m/z*: 290 (M+, 42%), 146 (69), 119 (100), 118 (25), 91 (25), 90 (63), 64 (21), 63 (21); ^1^H-NMR (DMSO-*d*_6_) δ: 12.61 (s, 1H, 3-H), 11.18 (s, 1H, amide-H), 8.19 (dd, *J* = 7.5 Hz, 0.8 Hz, 1H, 5-H), 8.11–8.08 (BB′ part of an AA′BB′ system, 2H, phenyl 3′-H, 5′-H), 7.92–7.84 (m, 4H, 7-H, 8-H, phenyl 2′-H, 6′-H), 7.67–7.61 (m, 1H, 6-H); ^13^C-NMR (DMSO-*d*_6_) δ: 161.2, 158.8, 146.6, 145.7, 141.9, 134.7, 133.1, 128.3, 127.7, 126.2, 122.8, 120.7, 118.8, 106.4. HRMS (EI, 70 eV) *m/z* calcd. for C_16_H_10_N_4_O_2_ (M^+^): 290.0804. Found: 290.0812.

*General Procedure for the Alkylation of the Anilides ***4b**–**f**. To a solution/suspension of the anilide **4** (3 mmol) in DMF (20 mL) was added K_2_CO_3_ (455 mg, 3.3 mmol) and propargyl bromide (490 mg of an 80% solution in toluene, 3.3 mmol), and the mixture was stirred at room temperature for 24 h (in the case of **4e** and **4f**, the reaction time was 48 h and the reagents were added in two equal portions of 1.65 mmol, one at the beginning and another one after 24 h). The mixture was poured into water (200 mL) and it was exhaustively extracted with CH_2_Cl_2_. The combined extracts were washed with water and brine, dried over Na_2_SO_4_, and evaporated. The residue was triturated with a little CHCl_3_ and recrystallised from an appropriate solvent (see below). 

*N-(4-Chlorophenyl)-4-oxo-3-(prop-2-yn-1-yl)-3,4-dihydroquinazoline-2-carboxamide* (**5b**). Yield: 870 mg (86%), colourless crystals, mp 186–189 °C (CHCl_3_). IR (KBr): 3300, 3264, 3190, 3124, 3070, 3004, 2970, 1686, 1656, 1602, 1586, 1568, 1542, 1490, 1474, 1466, 1422, 1388, 1336, 1312, 1240, 1166, 1092, 1010, 946, 902, 828, 790, 768, 698, 650, 562, 516, 498 cm^−1^; MS (EI, 70 eV) *m/z*: 338 ([M−1]^+^, 38%), 336 ([M−1]^+^, 100), 308 (51), 302 (77), 155 (42), 129 (48), 119 (57), 90 (44); ^1^H-NMR (CDCl_3_) δ: 9.73 (s, 1H, amide-H), 8.35 (d, *J* = 8.1 Hz, 1H, 5-H), 7.87–7.81 (m, 1H, 7-H), 7.79–7.76 (m, 1H, 8‑H), 7.74–7.67 (BB′ part of an AA′BB′ system, 2H, phenyl 2′-H, 6′-H, shows NOE on irradiation at 9.73 ppm), 7.65–7.58 (m, 1H, 6-H, shows NOE on irradiation at 8.73 ppm), 7.41–7.35 (AA′ part of an AA′BB′ system, 2H, phenyl 3′-H, 5′-H), 5.58 (d, *J* = 2.4 Hz, 2H, CH_2_), 2.28 (t, *J* = 2.4 Hz, 1H, C≡CH); ^13^C-NMR (CDCl_3_) δ: 161.1, 158.1, 145.0, 144.8, 135.4, 135.0, 130.4, 129.2, 129.1, 127.7, 127.5, 121.7, 121.3, 78.8, 72.0, 33.6. HRMS (EI, 70 eV) *m/z* calcd. for C_18_H_11_ClN_3_O_2_ ([M−H]^+^): 338.0696. Found: 338.0703. 

*N-(2-Methylphenyl)-4-oxo-3-(prop-2-yn-1-yl)-3,4-dihydroquinazoline-2-carboxamide* (**5c**). Yield: 724 mg (76%), colourless crystals, mp 208–210 °C (CHCl_3_). IR (KBr): 3230, 3058, 2922, 1680, 1660, 1600, 1544, 1476, 1458, 1424, 1386, 1326, 1310, 1240, 1172, 1046, 1026, 976, 948, 914, 858, 810, 772, 744, 692, 622, 592, 546, 502 cm^−1^; MS (EI, 70 eV) *m/z*: 317 (M^+^, 19%), 316 (36), 303 (21), 302 (100), 289 (24), 288 (45), 144 (28), 129 (30), 119 (37); ^1^H-NMR (CDCl_3_) δ: 9.69 (s, 1H, amide-H, shows NOE on irradiation at 2.44 ppm), 8.37 (d, *J* = 7.5 Hz, 1H, 5-H, shows NOE on irradiation at 7.64–7.59 ppm), 8.09 (d, *J* = 8.1 Hz, 1H, phenyl 6′-H, shows NOE on irradiation at 9.69 ppm), 7.87–7.82 (m, 1H, 7-H, shows NOE on irradiation at 7.64-7.59 ppm), 7.77 (d, *J* = 7.5 Hz, 1H, 8-H), 7.64–7.59 (m, 1H, 6-H, shows NOE on irradiation at 8.37 ppm), 7.32–7.26 (m, 2H, phenyl 5′-H, 3′-H, shows NOE on irradiation at 2.44 ppm, on irradiation at 8.09 ppm, or on irradiation at 7.18–7.13 ppm), 7.18–7.13 (m, 1H, phenyl 4′-H), 5.62 (d, *J* = 2.4 Hz, 2H, CH_2_), 2.44 (s, 3H, CH_3_, shows NOE on irradiation at 9.69 ppm), 2.28 (t, *J* = 2.4 Hz, 1H, C≡CH); ^13^C-NMR (CDCl_3_) δ: 161.2, 158.3, 145.5, 144.9, 134.9, 134.8, 130.6, 129.0, 128.9, 127.8, 127.5, 126.9, 125.7, 122.1, 121.7, 78.9, 71.9, 33.5, 17.7. HRMS (EI, 70 eV) *m/z* calcd. for C_19_H_16_N_3_O_2_ ([M+H]^+^): 318.1243. Found: 318.1238.

*N-(3,5-Dimethoxyphenyl)-4-oxo-3-(prop-2-yn-1-yl)-3,4-dihydroquinazoline-2-carboxamide* (**5d**). Yield: 885 mg (81%), colourless crystals, mp 208–210 °C (ethyl acetate). IR (KBr): 3336, 3260, 3044, 2974, 2940, 2840, 1696, 1602, 1534, 1464, 1408, 1382, 1360, 1332, 1298, 1242, 1202, 1154, 1064, 1034, 972, 956, 938, 896, 844, 810, 768, 730, 694, 678, 636, 582, 522, 494 cm^−1^; MS (EI, 70 eV) *m/z*: 364 (19%), 363 (78, M^+^), 362 (68), 348 (33), 335 (45), 332 (100), 320 (34), 190 (61), 184 (62), 156 (42), 155 (53), 145 (46), 129 (69), 119 (89), 102 (31), 90 (44); ^1^H-NMR (CDCl_3_) δ: 9.60 (s, 1H, amide-H), 8.35 (d, *J* = 7.8 Hz, 1H, 5-H), 7.87–7.77 (m, 2H, 7-H, 8-H), 7.61 (t, *J* = 7.2 Hz, 1H, 6-H), 6.95 (d, *J* = 1.8 Hz, 2H, phenyl 2′-H, 6′-H), 6.33 (t, unresolved, 1H, phenyl 4′-H), 5.59 (d, *J* = 2.1 Hz, 2H, CH_2_), 3.84 (s, 6H, OCH_3_), 2.28 (t, *J* = 2.1 Hz, 1H, C≡CH); ^13^C-NMR (CDCl_3_) δ: 161.2, 158.1, 145.4, 144.9, 138.5, 134.9, 128.9, 127.7, 127.5, 121.7, 98.6, 97.6, 78.8, 72.0, 55.5, 33.6. HRMS (EI, 70 eV) *m/z* calcd. for C_20_H_18_N_3_O_4_ ([M+H]^+^): 364.1297. Found: 364.1303.

*N-(4-Nitrophenyl)-4-oxo-3-(prop-2-yn-1-yl)-3,4-dihydroquinazoline-2-carboxamide* (**5e**). Yield: 565 mg (54%), colourless crystals, mp 204–206 °C (DMF/CHCl_3_). IR (KBr): 3298, 3270, 3158, 3094, 3008, 1696, 1664, 1614, 1586, 1556, 1512, 1426, 1340, 1306, 1248, 1166, 1110, 1042, 982, 946, 902, 856, 786, 750, 696, 648, 562, 520, 492 cm^−1^; MS (EI, 70 eV) *m/z*: 348 (M^+^, 41%), 347 (100), 319 (54), 301 (35), 155 (26), 129 (35), 119 (35), 90 (33); ^1^H-NMR (DMSO-*d*_6_) δ: 11.72 (s, 1H, amide-H), 8.32–8.28 (BB′ part of an AA′BB′ system, 2H, phenyl 3′-H, 5′-H), 8.25–8.22 (m, 1H, 5-H), 8.06–8.00 (AA′ part of an AA′BB′ system, 2H, phenyl 2′-H, 6′-H), 7.99–7.92 (m, 1H, 7-H), 7.86 (d, *J* = 7.8 Hz, 1H, 8-H), 7.72–7.66 (m, 1H, 6-H), 5.06 (d, *J* = 2.4 Hz, 2H, CH_2_), 2.50 (overlapped by solvent signal, 1H, C≡CH); ^13^C-NMR (DMSO-*d*_6_) δ: 160.0, 159.7, 147.5, 145.4, 143.8, 143.3, 135.3, 128.9, 127.7, 126.6, 124.9, 121.0, 120.2, 78.3, 75.2, 33.1. HRMS (EI, 70 eV) *m/z* calcd. for C_18_H_11_N_4_O_4_ ([M−H]^+^): 347.0780. Found: 347.0791.

*N-(4-Cyanophenyl)-4-oxo-3-(prop-2-yn-1-yl)-3,4-dihydroquinazoline-2-carboxamide* (**5f**). Yield: 765 mg (78%), colourless crystals, mp 199–201 °C (CHCl_3_). IR (KBr): 3300, 3258, 3180, 3108, 3058, 2998, 2224, 1692, 1660, 1598, 1536, 1508, 1474, 1432, 1408, 1390, 1318, 1246, 1230, 1176, 1164, 1110, 1042, 984, 948, 902, 840, 792, 770, 708, 694, 646, 550, 526, 508 cm^−1^; MS (EI, 70 eV) *m/z*: 328 (M+, 33%), 327 (100), 299 (45), 155 (15), 129 (17), 119 (22), 102 (18), 90 (16); ^1^H-NMR (DMSO-*d*_6_) δ: 11.56 (s, 1H, amide-H), 8.24 (d, *J* = 7.8 Hz, 1H, 5-H), 8.00–7.82 (m, 6H, 7-H, 8-H, phenyl 2′-H, 3′-H, 5′‑H, 6′-H), 7.69 (t, *J* = 7.6 Hz, 1H, 6-H), 5.05 (d, *J* = 1.2 Hz, 2H, CH_2_), 2.50 (overlapped by solvent signal, 1H, C≡CH); ^13^C-NMR (DMSO-*d*_6_) δ: 160.0, 159.7, 147.7, 145.5, 142.0, 135.3, 133.4, 128.8, 127.7, 126.6, 121.1, 120.4, 118.8, 106.6, 78.3, 75.2, 33.1. HRMS (EI, 70 eV) *m/z* calcd. for C_19_H_11_N_4_O_2_ ([M-H]^+^): 327.0892. Found: 327.0884.

*General Procedure for the Synthesis of the Substituted Luotonin A Derivatives ***8b**–**f**. To a solution of triphenylphosphine oxide (835 mg, 3 mmol) in dry CH_2_Cl_2_ (22 mL) was dropwise added trifluoro­methanesulfonic anhydride (0.25 mL, 1.5 mmol) at 0 °C under argon, and the mixture was stirred at the same temperature for 15 min. Then, the educt **5** (1 mmol) was added in one portion at 0 °C, and the mixture was stirred for 1 h (for **8e**: 24 h) while slowly warming to room temperature. Work-up for compounds **8b**, **8c**, **8d**, and **8f** (for **8e**, see below): the reaction was quenched by addition of 10% aqueous NaHCO_3_ (15 mL). The phases were separated and the aqueous layer was exhaustively extracted with CH_2_Cl_2_. The combined organic layers were washed with water and brine, dried (Na_2_SO_4_) and evaporated. The residue was triturated with CHCl_3_ and filtered; the crude material thus obtained was recrystallised from the appropriate solvent. 

*2-Chloroquinolino[2',3':3,4]pyrrolo[2,1-b]quinazolin-11(13H)-one (2-Chloroluotonin A)* [19] (**8b**). Yield: 313 mg (98%), pale yellow needles, mp > 330 °C decomp. (CHCl_3_) (lit.[19] mp 233–234 °C). IR (KBr): 3082, 3064, 2964, 1674, 1630, 1604, 1488, 1466, 1388, 1352, 1232, 1168, 1140, 1072, 1028, 928, 902, 836, 766, 738, 690, 662, 600, 524, 478 cm^−1^; MS (EI, 70 eV) *m/z*: 321 (M^+^, 28%), 320 (21), 319 (M^+^, 100), 284 (20), 77 (20), 76 (18), 63 (17), 50 (18); ^1^H-NMR (CDCl_3_) δ: 8.43 (dd, *J* = 9.0 Hz, 0.9 Hz, 1H, 10-H, shows NOE on irradiation at 7.63–7.57 ppm), 8.41 (d, *J* = 9.0 Hz, 1H, 4-H, shows NOE on irradiation at 7.79 ppm), 8.38 (s, 1H, 14-H, shows NOE on irradiation at 5.36 ppm and at 7.95 ppm), 8.12 (dd, *J* = 8.1 Hz, 0.6 Hz, 1H, 7-H), 7.95 (d, *J* = 2.4 Hz, 1H, 1-H), 7.90–7.84 (m, 1H, 8‑H, shows NOE on irradiation at 8.12 ppm, at 8.12 ppm, or at 7.63–7.57 ppm), 7.79 (dd, *J* = 9.0 Hz, 2.4 Hz, 1H, 3-H), 7.63–7.57 (m, 1H, 9-H), 5.36 (s, 2H, CH_2_); ^13^C-NMR (CDCl_3_) δ: 155.2, 149.3, 147.9, 134.7, 133.0, 132.2, 131.8, 130.6, 130.4, 129.3, 128.8, 127.6, 126.6, 126.5, 121.9, 121.4, 47.3.

*4-Methylquinolino[2',3':3,4]pyrrolo[2,1-b]quinazolin-11(13H)-one (4-Methylluotonin A)* [11] (**8c**). Yield: 245 mg (82%), colourless needles, mp 286–288 °C (CHCl_3_) (lit. [11] mp 294–295 °C). IR (KBr): 3516, 3446, 3064, 2918, 1680, 1628, 1606, 1500, 1464, 1442, 1376, 1348, 1316, 1276, 1234, 1184, 1158, 1106, 1026, 908, 774, 760, 692, 664, 648, 508 cm^−1^; MS (EI, 70 eV) *m/z*: 300 ([M+1]^+^, 19%), 299 (M^+^, 100), 284 (6), 150 (7), 135 (7), 122 (7); ^1^H-NMR (CDCl_3_) δ: 8.41 (dd, *J* = 7.8 Hz, 1.5 Hz, 1H, 10-H), 8.38 (s, 1H, 14-H, shows NOE on irradiation at 5.31 ppm or at 7.76 ppm), 8.07 (dd, *J* = 8.1 Hz, 0.6 Hz, 1H, 7-H, shows NOE on irradiation at 7.87–7.81 ppm), 7.87–7.81 (m, 1H, 8-H, shows NOE on irradiation at 8.07 ppm), 7.76 (d, *J* = 8.1 Hz, 1H, 1-H), 7.68 (d, *J* = 8.1 Hz, 1H, 3-H, shows NOE on irradiation at 3.03 ppm), 7.59–7.53 (m, 2H, 2-H, 9-H, shows NOE on irradiation at 7.76 ppm or at 7.87–7.81 ppm), 5.31 (s, 2H, CH_2_), 3.03 (s, 3H, CH_3_); ^13^C-NMR (CDCl_3_) δ: 160.7, 152.9, 150.2, 149.4, 148.7, 139.0, 134.4, 131.6, 130.7, 129.3, 128.8, 128.4, 127.3, 126.4, 125.9, 121.3, 47.2, 18.4. HRMS (EI, 70 eV) *m/z* calcd. for C_19_H_13_N_3_O (M^+^): 299.1059. Found: 299.1053.

*1,3-Dimethoxyquinolino[2',3':3,4]pyrrolo[2,1-b]quinazolin-11(13H)-one (1,3-Dimethoxyluotonin A)* (**8d**). Yield: 290 mg (84%), pale yellow needles, mp 295–297 °C (CHCl_3_). IR (KBr): 3066, 3016, 2940, 2838, 1680, 1624, 1580, 1504, 1466, 1408, 1392, 1366, 1332, 1302, 1256, 1212, 1154, 1144, 1094, 1042, 1020, 902, 830, 776, 692, 660, 586, 526 cm^−1^; MS (EI, 70 eV) *m/z*: 346 ([M+1]^+^, 22%), 345 (M^+^, 100), 331 (7), 330 (33), 287 (15), 259 (10), 173 (9), 129 (7); ^1^H-NMR (CDCl_3_) δ: 8.73 (s, 1H, 14-H, shows NOE on irradiation at 5.27 ppm), 8.42 (d, *J* = 8.1 Hz, 1H, 10-H), 8.10 (d, *J* = 7.8 Hz, 1H, 7-H), 7.87–7.81 (m, 1H, 8-H), 7.59–7.54 (m, 1H, 9-H), 7.37 (d, *J* = 1.5 Hz, 1H, 4-H), 6.60 (d, *J* = 2.4 Hz, 1H, 2-H), 5.27 (s, 2H, CH_2_), 4.01 (s, 3H, 1-OCH_3_, shows NOE on irradiation at 8.73 ppm), 3.98 (s, 3H, 3-OCH_3_, shows NOE on irradiation at 7.37 ppm); ^13^C-NMR (CDCl_3_) δ: 162.2, 160.7, 155.8, 152.9, 151.8, 151.5, 149.5, 134.4, 128.7, 127.2, 126.9, 126.5, 126.4, 121.3, 117.9, 100.6, 100.0, 56.0, 55.8, 47.5. HRMS (EI, 70 eV) *m/z* calcd. for C_20_H_16_N_3_O_3_ ([M+H]^+^): 346.1192, Found: 346.1190.

*2-Nitroquinolino[2',3':3,4]pyrrolo[2,1-b]quinazolin-11(13H)-one (2-Nitroluotonin A)* (**8e**). The reaction mixture was evaporated under reduced pressure and the residue was treated with 10% aqueous NaHCO_3_ (5 mL), followed by dilution with water (100 mL). The mixture was stirred at room temperature for 1 h, then the solid was collected by filtration, washed with water and dried. The material was taken up in CHCl_3_ (10 mL) and it was briefly refluxed. The suspension was filtered while hot and the solid was washed with hot CHCl_3_ and dried to afford 230 mg (69%) of **8e** as colourless crystals, mp > 330 °C decomp. (DMF). IR (KBr): 3068, 3006, 2938, 1674, 1630, 1606, 1578, 1528, 1490, 1464, 1404, 1344, 1300, 1234, 1174, 1140, 1084, 942, 906, 856, 828, 774, 734, 692, 654, 598, 536, 486, 466 cm^−1^; MS (EI, 70 eV) *m/z*: 331 ([M+1]^+^, 20%), 330 (M^+^, 100), 284 (72), 272 (19), 128 (18), 77 (21), 76 (19), 63 (17); ^1^H-NMR (DMSO-*d*_6_) δ: 9.20 (s, 1H, 1-H), 9.05 (s, 1H, 14-H), 8.54 (d, *J* = 9.0 Hz, 1H, 3-H), 8.44 (d, *J* = 9.0 Hz, 1H, 4-H), 8.27 (d, *J* = 7.5 Hz, 1H, 10-H), 7.99–7.88 (m, 2H, 7-H, 8-H), 7.64 (t, unresolved, 1H, 9-H), 5.35 (s, 2H, CH_2_); ^13^C-NMR (DMSO-*d*_6_) δ: 159.5, 155.1, 152.4, 150.1, 148.8, 145.9, 134.6, 134.1, 132.7, 131.5, 128.2, 127.6, 127.5, 125.9, 125.4, 123.5, 121.2, 47.6. HRMS (EI, 70 eV) *m/z* calcd. for C_18_H_10_N_4_O_3_ (M^+^): 330.0753. Found: 330.0751.

*11-Oxo-11,13-dihydroquinolino[2',3':3,4]pyrrolo[2,1-b]quinazoline-2-carbonitrile (2-Cyanoluotonin A)* (**8f**). Yield: 168 mg (54%), colourless crystals, mp > 330 °C decomp. (CHCl_3_). IR (KBr): 3058, 3004, 2954, 2226, 1676, 1628, 1604, 1498, 1464, 1438, 1410, 1372, 1354, 1334, 1238, 1182, 1152, 1028, 932, 906, 832, 778, 692, 656, 598, 528, 482 cm^−1^; MS (EI, 70 eV) *m/z*: 311 ([M+1]^+^, 16%), 310 (M^+^, 100), 282 (12), 281 (7), 254 (7), 141 (5); ^1^H-NMR (DMSO-*d*_6_) δ: 8.87 (s, 1H, 14-H), 8.86 (d, *J* = 1.8 Hz, 1H, 1-H), 8.41 (d, *J* = 8.7 Hz, 1H, 4-H), 8.30 (d, *J* = 7.8 Hz, 1H, 10-H), 8.17 (dd, *J* = 8.7 Hz, 1.8 Hz, 1H, 3-H), 7.96–7.93 (m, 2H, 7-H, 8-H), 7.68–7.62 (m, 1H, 9-H), 5.35 (s, 2H, CH_2_); ^13^C-NMR (DMSO-*d*_6_) δ: 159.6, 154.5, 152.5, 149.1, 148.9, 135.3, 134.6, 132.7, 132.6, 131.1, 130.9, 128.2, 127.7, 127.5, 125.9, 121.2, 118.4, 110.5, 47.7. HRMS (EI, 70 eV) *m/z* calcd. for C_19_H_10_N_4_O (M^+^): 310.0855. Found: 310.0862.

*4-Oxo-N-(pyridin-4-yl)-3,4-dihydroquinazoline-2-carboxamide* (**9**). To a solution of 4-aminopyridine (753 mg, 8 mmol) in dry 1,2-dichloroethane (20 mL) under argon was added dropwise a 2 M solution of AlMe_3_ (4.0 mL, 8 mmol) in heptane. The mixture was stirred for 30 min at room temperature, then the ester **1** (1.091 g, 5 mmol) was added in one portion, and the mixture was refluxed for 2 h. After cooling to 0 °C, it was then quenched by slow addition of 2 N HCl (20 mL), followed by water (80 mL). The two clear phases were separated and the aqueous phase was brought to pH 6–7 by addition of 2 N ammonia under ice-cooling. The resulting suspension was kept in the refrigerator overnight, then the product was collected by filtration, washed with water and dried to afford 960 mg (72%) of **9** as colourless crystals, mp 304–306 °C (EtOH). MS (EI, 70 eV) *m/z*: 266 (M^+^, 68%), 238 (26), 146 (70), 121 (77), 119 (100), 118 (20), 90 (38), 64 (18), 63 (17), 51 (17); ^1^H-NMR (DMSO-*d*_6_) δ: 12.59 (br, 1H, 3-H), 11.12 (br, 1H, amide-H), 8.60–8.48 (m, unresolved, 2H, pyridine 2′-H, 6′-H), 8.20 (d, *J* = 7.8 Hz, 1H, 5-H), 7.97–7.88 (m, 4H, 7-H, 8-H, pyridine 3′-H, 5′-H, shows NOE on irradiation at 11.12 ppm or at 8.60–8.48 ppm), 7.68–7.62 (m, 1H, 6-H, shows NOE on irradiation at 8.20 ppm); ^13^C-NMR (DMSO-*d*_6_) δ: 160.9, 159.1, 150.3, 146.7, 145.4, 144.5, 134.8, 128.4, 127.9, 126.2, 122.8, 114.4. Anal. calcd. for C_14_H_10_N_4_O_2_**^.^** 0.5 H_2_O: C, 61.09; H, 4.03; N, 20.35. Found: C, 61.08; H, 3.73; N, 20.20.

*4-Oxo-3-(prop-2-yn-1-yl)-N-(pyridin-4-yl)-3,4-dihydroquinazoline-2-carboxamide* (**10**). To a suspension of **9** (666 mg, 2.5 mmol) in DMF (15 mL) wass added K_2_CO_3_ (414 mg, 3 mmol) and propargyl bromide (446 mg of an 80% solution in toluene, 3 mmol), and the mixture was stirred at room temperature for 24 h. It was then poured into water (50 mL) and it was exhaustively extracted with CH_2_Cl_2_. The combined extracts were washed with water and brine, dried over Na_2_SO_4_, and evaporated. The residue was purified by column chromatography (CH_2_Cl_2_/MeOH, 19+1), followed by recrystallisation from ethyl acetatre/light petroleum to give 365 mg (48%) of **10** as colourless crystals, mp 183–185 °C. MS (EI, 70 eV) *m/z*: 304 (M^+^, 31%), 303 (100), 275 (58), 155 (35), 145 (23), 131 (34), 129 (44), 121 (32), 119 (37), 90 (27), 78 (28), 57 (33), 51 (53); ^1^H-NMR (CDCl_3_) δ: 10.02 (br, 1H, amide-H), 8.60 (d, *J* = 5.7 Hz, 2H, pyridine 2′-H, 6′-H), 8.34 (dd, *J* = 6.9 Hz, 0.9 Hz, 1H, 5-H), 7.86–7.81 (m, 1H, 7-H), 7.76 (d, *J* = 7.5 Hz, 1H, 8-H), 7.68 (d, *J* = 5.7 Hz, 2H, pyridine 3′-H, 5′-H), 7.64–7.58 (m, 1H, 6-H), 5.55 (d, *J* = 2.4 Hz, 2H, CH_2_), 2.28 (t, *J* = 2.4 Hz, 1H, C≡CH); ^13^C-NMR (CDCl_3_) δ: 160.9, 158.7, 150.9, 144.6, 144.5, 143.8, 135.0, 129.3, 127.7, 127.5, 121.8, 113.9, 78.6, 72.2, 33.5. HRMS (EI, 70 eV) *m/z* calcd. for C_17_H_12_N_4_O_2_ (M^+^): 304.0960. Found: 304.0953.

*4-Oxo-N-(2-thienyl)-3,4-dihydroquinazoline-2-carboxamide* (**11**). To an ice-cooled solution of *tert*-butyl *N*-(2-thienyl)carbamate [14,26] (1.995 g, 10 mmol) in CH_2_Cl_2_ (60 mL) was added trifluoroacetic acid (15 mL), then the cooling bath was removed and the solution was stirred for 2 h. It was then slowly added into a well-stirred, ice-cooled mixture of CH_2_Cl_2_ (200 mL) and a solution of Na_2_CO_3_ (22.1 g, 0.2 mol) in water (200 mL). The phases were separated and the aqueous layer was extracted with CH_2_Cl_2_ (2 × 100 mL). The combined organic phases were washed with brine, dried over Na_2_SO_4_ and filtered. To this solution was added 1,2-dichloroethane (30 mL) and it was then concentrated under reduced pressure to approx. 25–30 mL (max. bath temperature 20 °C). This solution was transferred into a two-necked flask equipped with a rubber septum and a reflux condenser. Under argon atmosphere, a 2 M solution of AlMe_3_ (4.0 mL, 8 mmol) in heptane was added dropwise at room temperature, and stirring was continued for 30 min. The ester **1** (1.091 g, 5 mmol) was added in one portion, and the mixture was refluxed for 2 h. After cooling to 0 °C, it was then quenched by slow addition of 2 N HCl (20 mL), followed by water (80 mL). The phases were separated and the aqueous layer was extracted with CH_2_Cl_2_ (3 × 100 mL). The combined organic layers were washed with water, dried over Na_2_SO_4_ and evaporated. The residue was purified by column chromatography (CH_2_Cl_2_/ethyl acetate, 6+1) to give 1.29 g (95%) of **11** as a yellow solid, mp 243–246 °C. MS (EI, 70 eV) *m/z*: 271 (M^+^, 12%), 211 (15), 210 (100), 119 (16), 117 (19), 91 (75), 77 (19), 57 (16); ^1^H-NMR (DMSO-*d*_6_) δ: 12.53 (br, 1H, 3-H), 12.19 (br, 1H, amide-H), 8.20 (d, *J* = 8.1 Hz, 1H, 5-H), 7.94–7.86 (m, 2H, 7-H, 8‑H), 7.66–7.60 (m, 1H, 6-H), 7.22 (d, *J* = 3.9 Hz, 1H, thiophene 3′-H), 7.11 (d, *J* = 4.8 Hz, 1H, thiophene 5′-H), 6.95 (t, *J* = 4.5 Hz, 1H, thiophene 4′-H); ^13^C-NMR (DMSO-*d*_6_) δ: 160.8, 156.4, 146.9, 145.2, 138.7, 134.7, 128.2, 127.8, 126.2, 124.4, 122.8, 118.5, 114.4. HRMS (EI, 70 eV) *m/z* calcd. for C_13_H_10_N_3_O_2_S ([M+H]^+^): 272.0494. Found: 272.0493.

*4-Oxo-3-(prop-2-yn-1-yl)-N-(2-thienyl)-3,4-dihydroquinazoline-2-carboxamide* (**12**). To a suspension of **11** (814 mg, 3 mmol) in DMF (20 mL) wass added K_2_CO_3_ (455 mg, 3.3 mmol) and propargyl bromide (490 mg of an 80% solution in toluene, 3.3 mmol), and the mixture was stirred at room temperature for 24 h. It was then poured into water (100 mL) and it was extracted with ethyl acetate (2 × 100 mL). The combined extracts were washed with water and brine, dried over Na_2_SO_4_, and evaporated. The residue was purified by column chromatography (CH_2_Cl_2_) to give 780 mg (84%) of **12** as a yellow solid, mp 208–210 °C. MS (EI, 70 eV) *m/z*: 309 (M^+^, 98%), 308 (55), 292 (16), 281 (50), 276 (57), 183 (37), 156 (47), 155 (68), 145 (47), 136 (64), 129 (81), 119 (100), 97 (46), 90 (71); ^1^H-NMR (CDCl_3_) δ: 10.41 (br, 1H, amide-H), 8.37 (dd, *J* = 8.2 Hz, 1.1 Hz, 1H, 5-H), 7.85 (td, *J* = 7.5 Hz, 1.3 Hz, 1H, 7-H), 7.80 (dd, *J* = 8.1 Hz, 1.0 Hz, 1H, 8-H), 7.62 (td, *J* = 7.5 Hz, 1.6 Hz, 1H, 6-H), 7.05–7.02 (m, 1H, thiophene 5′-H), 6.99–6.96 (m, 2H, thiophene 3′-H, 4′-H), 5.65 (d, *J* = 2.4 Hz, 2H, CH_2_), 2.26 (t, *J* = 2.4 Hz, 1H, C≡CH); ^13^C-NMR (CDCl_3_) δ: 161.1, 156.6, 144.8, 144.2, 137.9, 135.0, 129.1, 127.7, 127.5, 124.5, 121.8, 119.1, 113.9, 78.7, 72.0, 33.6. HRMS (EI, 70 eV) *m/z* calcd. for C_16_H_12_N_3_O_2_S ([M+H]^+^): 310.0650. Found: 310.0648.

*Thieno[3′′,2′′:5′,6′]pyrido[2′,3′:3,4]pyrrolo[2,1-b]quinazolin-7(5H)-one* (**13**). To a solution of triphenyl-phosphine oxide (835 mg, 3 mmol) in dry CH_2_Cl_2_ (22 mL) was dropwise added trifluoro­methanesulfonic anhydride (0.25 mL, 1.5 mmol) at 0 °C under argon, and the mixture was stirred at the same temperature for 15 min. Then, a suspension of **12** (309 mg, 1 mmol) in dry CH_2_Cl_2_ (20 mL) was added over a period of 10 min at 0 °C, and the mixture was stirred for 1 h while slowly warming to room temperature. The reaction was quenched by addition of 10% aqueous NaHCO_3_ (30 mL). The phases were separated and the aqueous layer was exhaustively extracted with CH_2_Cl_2_. The combined organic layers were washed with water and brine, dried (Na_2_SO_4_) and evaporated. The residue was purified by column chromatography (CH_2_Cl_2_/ethyl acetate, 6+1), followed by recrystallisation from EtOH to afford 160 mg (55%) of **13** as almost colourless needles, mp 318–321 °C. MS (EI, 70 eV) *m/z*: 292 (19%), 291 (100, M^+^), 290 (30), 263 (20), 262 (15), 236 (7), 235 (8), 146 (17), 131 (11), 118 (7), 105 (9), 77 (10); ^1^H-NMR (CDCl_3_) δ: 8.45–8.40 (m, 1H, 8-H), 8.37 (s, 1H, 4-H), 8.08–8.04 (m, 1H, 11-H), 7.88–7.82 (m, 1H, 10-H), 7.80 (d, *J* = 6.3 Hz, 1H, 2-H), 7.60–7.54 (m, 1H, 9-H), 7.42 (d, *J* = 6.3 Hz, 1H, 3-H), 5.29 (s, 2H, CH_2_); ^13^C-NMR (CDCl_3_) δ: 164.0, 160.6, 152.6, 149.4, 147.7, 134.6, 134.5, 131.3, 129.9, 128.5, 127.2, 126.4, 126.2, 121.3, 121.1, 47.4. Anal. calcd. for C_16_H_9_N_3_OS: C, 65.97; H, 3.11; N, 14.42. Found: C, 66.15; H, 3.29; N, 13.93. HRMS (EI, 70 eV) *m/z* calcd. for C_16_H_10_N_3_OS ([M+H]^+^): 292.0546. Found: 292.0550.

## 4. Conclusions

By applying Weinreb’s amidation method to the preparation of the anilide-type key intermediates, we achieved a significant improvement of Yao’s synthetic route to luotonin A and A-ring modified derivatives thereof in terms of overall yields (**8a**: 65%, **8b**: 66%, **8c**: 47%, **8d**: 50%, **8e**: 30%, **8f**: 33%; all isolated overall yields are based on commercially available anthranilamide as starting material), brevity and—most importantly—versatility with regard to the aromatic amine component, even if the latter has less favourable nucleophilicity, solubility and/or stability properties. This is demonstrated by the concise synthesis of a small library of luotonin A analogues, including a novel thiophene isoster of the alkaloid.
